# Caring for children and families during the COVID-19 pandemic: innovations and changes experienced by nurses*

**DOI:** 10.1590/1980-220X-reeusp-2023-0355en

**Published:** 2024-06-28

**Authors:** Talita Cristina Pegorin, Margareth Angelo

**Affiliations:** 1 Universidade de São Paulo, Escola de Enfermagem, Departamento de Enfermagem Materno-Infantil e Psiquiátrica, São Paulo, SP, Brazil.

**Keywords:** COVID-19, Pediatric Nursing, Family, Qualitative research, COVID-19, Enfermería Pediátrica, Familia, Investigación Cualitativa., COVID-19, Enfermagem Pediátrica, Família, Pesquisa Qualitativa

## Abstract

**Objective:**

To understand the experience of nurses working in pediatric units in the face of innovations and changes in the process of caring for children and families during the COVID-19 pandemic. Also, the objective is to understand the typical experience of nurses in this care.

**Method:**

Qualitative research, which involved the participation of 16 nurses from pediatric units of a public teaching hospital. The data were analyzed according to the theoretical-methodological framework of Alfred Schütz’s social phenomenology.

**Results:**

The participants’ reports generated the categories: the challenge of experiencing changes amid fear, the team’s adaptation to innovations and changes caused by the COVID-19 pandemic and the expectation for care and the work process.

**Conclusion:**

The understanding of the nurses’ experience highlighted changes, team adaptations and expectations for the care of children and families, which, although permeated by learning, were experienced by ethical dilemmas and moral suffering for these professionals.

## RESUMO

**Objetivo:**

Compreender a vivência do enfermeiro que atua em unidades pediátricas diante das inovações e das mudanças no processo de cuidar da criança e da família durante a pandemia de COVID-19 e apreender o típico da vivência dos enfermeiros neste cuidado.

**Método:**

Pesquisa qualitativa, que contou com a participação de 16 enfermeiros de unidades pediátricas de um hospital público e de ensino. Os dados foram analisados de acordo com o referencial teórico-metodológico da fenomenologia social de Alfred Schütz.

**Resultados:**

Os relatos dos participantes geraram as categorias: o desafio de vivenciar as mudanças em meio ao medo, a adaptação da equipe às inovações e às mudanças provocadas pela pandemia de COVID-19 e a expectativa para o cuidado e processo de trabalho.

**Conclusão:**

A compreensão da vivência dos enfermeiros evidenciou mudanças, adaptações da equipe e expectativas ao cuidado da criança e da família que, embora permeada por aprendizagem, foi vivenciada por dilemas éticos e sofrimento moral por esses profissionais.

## RESUMEN

**Objetivo:**

Comprender la vivencia del enfermero que actúa en unidades pediátricas frente a las innovaciones y los cambios en el proceso de cuidar al niño y su familia durante la pandemia de COVID-19 y aprehender lo típico de la vivencia de los enfermeros en este cuidado.

**Método:**

Se trata de una investigación cualitativa con la participación de 16 enfermeros de unidades pediátricas de un hospital público docente. Los datos se analizaron según el referencial teórico-metodológico de la fenomenología social de Alfred Schütz.

**Resultados:**

Los relatos de los participantes concibieron las siguientes categorías: el reto de experimentar el cambio en medio del miedo, la adaptación del equipo a las innovaciones y los cambios provocados por la pandemia COVID-19, y las expectativas sobre la atención y el proceso de trabajo.

**Conclusión:**

La comprensión de la experiencia de los enfermeros mostró cambios, adaptaciones del equipo y expectativas en la atención al niño y a su familia que, aunque impregnada de aprendizaje, fue vivida por estos profesionales como dilemas éticos y sufrimiento moral.

## INTRODUCTION

The new Coronavirus pandemic has brought challenges for professionals to provide patient- and family-centered care: an approach that aims at planning, participation and evaluation between family members, patients and health professionals^(1)^.

Among the challenges, the establishment of new policies for the movement of people in the hospital environment caused one of the most important changes for pediatric care. Differing between institutions, these policies became widely disseminated in these environments and significantly affected the presence of family members^(2)^.

As consequences of such policies, difficulties for family participation and presence in the hospital environment were established. Among them, the lack of understanding of the new scenario among family members, children and professionals^(3)^, difficulty in communication and involvement focused on the patient’s physical condition^(4)^, in addition to the limitation in the participation of family members in clinical care and their absence from bedside^(5,6)^.

With the impaired participation of these individuals during hospitalization and given the effects of separating the child from their family members, strategies have been described to safely overcome these difficulties, especially with measures that emphasize the use of virtual resources for care^(7,8)^. Although the strategies are useful, ethical challenges were established for nurses in the face of changes in the care scenario^(9)^. These changes refer to the safety of professionals, patients and their families, the allocation of resources and the relationships established with those they care for^(9)^.

Considering the described care scenario, understanding the experience of these professionals in the face of innovations in the process of caring for children and families during this period allowed, from the perspective of social phenomenology^(10)^, a unique understanding, given by a collective perspective of experiences established. Furthermore, understanding the innovations and changes in care for children and their families can demonstrate this context of major changes in the health area, including the areas of pediatric nursing and family nursing.

This study was guided by the following question: “How did nurses experience changes and innovations in the child and family care process during the COVID-19 pandemic?” Thus, the established objective is to understand the experience of nurses working in pediatric units in the face of innovations and changes in the process of caring for children and families during the COVID-19 pandemic and to understand the typical experience of nurses in this care.

## METHOD

### Study Design

Qualitative research based on Alfred Schütz’s social phenomenology.

### Theoretical-Methodological Framework

Alfred Schütz’s theory incorporates the author’s biographical experience and temporality as indispensable factors in understanding the genesis of his motivations for action. The changes and innovations established in pediatric care, especially in the health context of the COVID-19 pandemic, constituted aspects that permeated the nurses’ work experience, being an important phenomenon experienced by these professionals, as well as a motivation for choosing of social phenomenology. To understand this phenomenon, concepts such as: the life world, the natural attitude, intersubjectivity, the face-to-face relationship, the biographical situation, the knowledge background, the relevance system, social action and motivational conduct (reasons for and reasons why) were used^(10)^.

### Setting

The study was carried out in pediatric units – children’s emergency room, hospitalization ward and intensive and neonatal care wards of a public teaching and secondary hospital, located in the city of São Paulo.

### Population and Selection Criteria

Nurses who work in pediatrics and who have monitored, since the beginning of the COVID-19 pandemic, children’s and family care processes were included.

### Data Collection

Data collection was carried out through a phenomenological interview by one of the authors, with previous experience in qualitative research and a specialist in the field of pediatric nursing. Participants were invited by the nursing manager, followed by contact by the main researcher, who formalized it by email. The contact, by the main researcher, was possible because she was a collaborator at the institution prior to the research.

The interviews took place from April to July 2020 and were carried out online, with an average duration of thirty minutes. The videoconferencing feature was used to encourage interaction, however, only the audio of these sessions was recorded.

Obtaining participants’ reports was guided by questions based on the concept of human motivation in the adopted theoretical framework. The triggering question was “Tell me about the innovations and changes in the child and family care process during the COVID-19 pandemic. Data such as age, gender, training and time working at the site were also collected.

Eighteen participants were invited, however, only sixteen agreed to participate in the study. There was no participation from the participants to check the transcription of the interviews. To have greater representativeness of participants, it was necessary to use the snowball sampling strategy^(11)^, which allowed one participant to indicate another potential participant and so on. Collection was completed as the data was considered sufficient to understand the phenomenon investigated^(12)^.

### Data Analysis and Processing

To carry out the analysis of the participants’ reports, the steps described for phenomenological research were used^(13)^: careful reading of the participants’ reports, in order to identify and understand the global meaning of the experience lived by the research participants; selection of participants’ reports, with the intention of discriminating the units of meaning, which occurred as their relevance to the study phenomenon was observed; grouping of units, respecting their convergence of meanings, making it possible, at this stage, to compose categories representing the phenomenon experienced by the participants; synthesis of all units, integrating them with the concepts of social phenomenology and literature on the subject. This research met the Consolidated Criteria for Reporting Qualitative Research (COREQ)^(14)^.

### Ethical Aspects

This study respected the ethical guidelines established by Resolution 466/2012, of the National Health Council^(15)^, and was submitted to and approved by the Research Ethics Committee (REC) of the proposing institution and co-participating institution. Approval took place in 2021 under the respective numbers 4,582,098 and 4,614,009. For the nurses’ anonymity, the letter P (participants) was used followed by Arabic numbers. Participants signed the Free and Informed Consent Form (FICF) virtually.

## RESULTS

16 nurses participated in the study, aged between 38 and 55 years, 14 recognized themselves as female and 2 as male; 9 had specialized training in other and related areas, 3 at master’s level and 1 with a doctorate. The time these professionals have worked in the area ranged from 10 to 34 years. Regarding the area of activity, 5 nurses worked in pediatric and neonatal intensive care units, 6 in children’s emergency rooms and 5 in pediatric inpatient units.

Based on Alfred Schütz’s social phenomenology^(10),^ three categories were revealed: “The challenge of experiencing changes amid fear”, “Team adaptation to innovations and changes caused by the COVID-19 pandemic” and “Expectation for care and work process”.

### The Challenge of Experiencing Change Amid Fear

The experience of nurses working in pediatrics was marked by the changes that occurred in the hospital environment to combat COVID-19. Among them are structural adjustments, which were experienced through significant impact on the physical structure, human and material resources.

*(...) we were going to reduce beds and then we closed a wing and then didn’t open it again. And then COVID came, a COVID ward was used. The maternity ward was in our other wing* (P1).

*(...) I work in an emergency room and there was a very important structural change here, which was the construction of a sector called “gripário*”(P3).

Changes in the flow of care were also highlighted by nurses, with emphasis on the family’s marathon search for care and the child’s admission with a focus on COVID-19 in light of the new care adjustments, which restricted care and imposed isolation measures.

*(...) with COVID, it was restricted, they didn’t even attend, the family’s advice was not to go to the hospital. There were mothers who went to 3.4 hospitals for their child to be treated* (P1).

*(...) All children go through the same isolation process. This is even a stress factor among us, because we don’t agree, we don’t need to generalize* (P6).

In their experiences, nurses also highlighted the restrictions on the presence of visitors and companions as changes, which impacted the care process and the relationships between family members and the hospitalized child.

*(...) If it was done one way before, changing companions, now it’s done another way. The issue of visits, which I think makes a lot of difference for children* (P2).

*(...) The first change was big and negative, we had to restrict access to father and mother, which was something we normally did* (P10).

The adjustment in carrying out practices and procedures, followed by changes in clothing, was a transformation highlighted by the participants, thus imposing strict control of care practices not only for patients, but for themselves and other team members.

*(...) In terms of exposure, we have a new way of taking care of ourselves, right? It was a new way of working that we had to learn how to do* (P3).

The restrictive visitation and monitoring routine and COVID-19 tracking flow for children and families were difficulties brought about by the changes, aspects experienced by these professionals.

*(...) It’s a challenge, because often the mother doesn’t understand, the child sometimes doesn’t understand either. The family doesn’t understand* (P7).

*(...) But as for family, it’s difficult, you know? You have to mention that only she can be a companion, that she will not be able to receive visitors* (P12).

Impasses with initial visitation routines, as well as ethical challenges with the restriction of visits, resulted in difficulties for nurses to perform their role with regard to a child- and family-centered approach.

*(...) we were doing well at the beginning of the pandemic and then what happened: the institution released the mother, not the father. But the mother was hospitalized, and who saw this child? nobody? We couldn’t release the father, so that was very difficult* (P6).

*(...) the patient is hospitalized with COVID, she will not be able to stay with a companion. This does not apply to the child. It is an infringement of the child’s rights too* (P3).

Difficulty working with the new organization of the units and obstacles with the use of attire required nurses to make continuous adjustments in their work process to make it closer to what they consider to be humanized care.

*(...) working within a pediatric “flu” is quite challenging. I needed to change my way of relating to colleagues and patients and their families* (P3).

*(...) I think that the mask greatly distorts this reception of care* (P2).

Fear, lack of knowledge and greater exposure to the virus were experienced by the nurses in this study. The nurses described fear of being more exposed to the virus, being closer to the child and family, being in a risky hospital environment, witnessing the illness of family members and colleagues, reinforcing the feeling of vulnerability in the environment.

*(...) we were feeling extremely exposed, something that no one knew about. And then our teammates started to catch COVID and got sick* (P1).

*(...) the family also exposes us as professionals* (P6).

Lack of knowledge, fear and insecurity when experiencing the beginning of the pandemic, due to misinformation about the disease and forms of protection, were aspects described by nurses, which draw attention to the impact and insecurity in relationships between peers.

*(...) So, all that attire that we had to do, whether we were doing it right or whether we were doing it wrong* (P8).

*(...) everyone has a certain degree or another of stress, right? everyone is* (P10).

The nurse, faced with the lack of knowledge about the disease, especially in the early stages, was more afraid of infecting others than himself – especially fear of contaminating his family.

*(...) for me, it’s funny, I wasn’t so afraid of getting infected by the disease, but I was terrified of infecting someone, for example my family* (P14).

This professional understands that the risk of exposure to the virus had an impact on care, in addition to the loss of physical contact with peers, this feeling was present in the relationships between family members and hospitalized children and families.

*(...) I didn’t see my parents for a long time and I was really afraid of passing COVID on to them, so it was kind of painful* (P1).

*(...) the issue of hugs, there was often this, you know, in everyday life, to comfort the family, and now you need to keep a distance, I think this distance from hospitalized families was very bad* (P5).

The feeling of fear, lack of knowledge and greater exposure to the virus, explicit in the initial phases of the pandemic, became less intense after the vaccination of health professionals, as nurses began to feel less worried about being vaccinated and working with children who were not infected.

*(...) we started vaccinating, I vaccinated, so I felt relief in that part, we have to have a little peace of mind to calm the team down too* (P14).

*(...) Later, when we saw that things weren’t like that, also due to the fact that the child didn’t have so many cases, I think this was a very reassuring factor* (P6).

Through self-care and peer support, participants in this study sought coping strategies, with emphasis on the mental health of professionals*.*

*(...) we are having to take care of our mental health, they (institution) have been guiding us* (P1).

*(...) worry about the health of the professional too, so take more care of ourselves* (P3).

### Team Adaptation to Innovations and Changes Caused by the Covid-19 Pandemic

Throughout the pandemic, participants experienced adaptation and adjustment to new ways of caring for children and families. To do this, they experienced new restrictive visitation and monitoring routines, the introduction of a new COVID-19 tracking flow and the use of new materials.

*(...) Before we had this: “we can’t come, uncle, we can’t come...” So, all these routine things in the sector, we created as things went along* (P3).

*(...) we were allowed to collect the exams from these parents at the institution, before it took a week for the results to be ready, now the parents can return to stay with the child* (P6).

*(...) We needed to learn how to manipulate other respiratory resources that are more resources in cases of COVID, for example* (P3).

During this time, nurses recognized an attitude of adaptation, which was possible due to the current pandemic and their feeling of adaptation to new work processes.

*(...) speaking specifically about the pandemic, I think the worst part is over* (P10).

*(...) the team had to adapt in relation to assistance and the famil*y(P16).

*(...) it’s a question of adaptation, I’ve already adapted, families have also adapted* (P6).

The need to innovate in the ways of providing care was experienced. The nurses understood that they had to reinvent themselves looking for alternatives so that children not only had access to toys but could also communicate.

*(...) Excessive care with the hygiene of these toys, when we put them in isolation, right, so we have to be very careful* (P4).

*(...) So it’s logical that we had to learn to talk through our eyes, right?* (P2).

*(...) before, cell phone use was very restricted. Now we free up our cell phones to chat via video. This was not allowed, but we do it to try to alleviate this separation* (P11).

In addition to these aspects, it was important for nurses to make visiting and monitoring routines more flexible in some situations.

*(...) sometimes children need intervention in the ICU, at that moment, it is very important for the couple to be there, to provide support for each other and for the child as well* (P7).

*(...) we did training (qualification) and I ended up allowing my mother and sister to help with the training, something that at the beginning we were unable to release* (P13).

By making these new institutional routines more flexible, nurses had implications for the work process. To do this, they recognize the risks of transmitting the virus and have had conflicts with team members and other family members.

*(...) I tried to adapt to the dynamics of that family. On the other hand, I also see that perhaps this had an impact when you went to shift changes and your colleague questioned you* (P15).

*(...) we had several conflicts regarding this, because the mother complains that we allowed the father to stay, because the child goes to the ICU, and she doesn’t understand...* (P16).

The nurse understands that he or she evaluates and makes these institutional routines more flexible because he puts himself in the family’s shoes.

*(...) I was on the other side, right? on the patient side* (P1).

*(...) For me it is very difficult because I consider myself a very empathetic, very human person, and I see the need for families* (P6).

Faced with the changes brought about by the pandemic, participants developed learnings, which involved sharing experiences, caring for their families and the effective use of safety measures.

*(...) listening to other points of view that are different from yours, agreeing, disagreeing, all of this is very rich* (P10).

*(...) everything that happened during the pandemic were many factors that made us reevaluate the form of care not only for the child but for the family* (P13).

*(...) we learned to really take care in the hospital so that we don’t take it home, for me it was one of the biggest learnings* (P4).

### Expectation for Care and Work Process

The experience of nurses who work in pediatrics is also demarcated by their expectations. When uncertain about the end of the pandemic, this professional makes projections amidst the doubt that these expectations will happen.

*(...) I believe it will continue this way, because the pandemic is far from over and so I think that we will continue with this restriction of people* (P6).

Among the expectations, the desire for safe measures and appreciation of the work carried out were significant points so that this professional could continue with his role, at least feeling protected and valued.

*(...) Regarding the mask, I think we will wear it for a long time, it is really protective for us* (P14).

*(...) And in terms of clothing, I think it was very positive* (P15).

*(...) After the pandemic, I think this is it, a positive thing, in the sense of valuing our work as professionals* (P13).

As can be seen, the professional also makes projections for the child and the family, when believing that there has been harm to the care, as a result of the health context, the nurse aims for care provided through creative, humane and child-centered measures and in the family.

*(...) My expectation is that the scenario will improve, that we will see innovations in the human scenario. I think we need to think about more humane solutions* (P8).

*(...) That children continue to be in the presence of their families, that this separation does not occur. Because we had already achieved many things, I’m not talking about us as an emergency room team, I’m talking about this at the level of other institutions* (P15).

### Typical Lived

The categories from the nurses’ reports made it possible to understand the care of children and families during the COVID-19 pandemic and to understand the typical experiences of these professionals, such as: those who faced the challenge of experiencing changes in the environment to fear, welcoming the changes that occurred in the hospital environment, the difficulties faced; fear, lack of knowledge and greater exposure to the virus; the search for coping strategies to continue with care; who led the team’s adaptation to the innovations and changes caused by the COVID-19 pandemic, with the adjustment to new ways of caring for children and families, with an adaptive stance, with continuous reinforcement of the need to innovate ways of providing care, and the understanding that the changes resulting from the pandemic brought learning; who has expectations for the care and work process, amid the uncertainty of the end of the pandemic, has a desire for safe measures and appreciation of the work carried out and who projects creative, humane and child- and family-centered measures.

## DISCUSSION

The reports of nurses who work in pediatrics revealed, through the recurrence and convergence of their statements, a common world. This collective construction made it possible to identify the experiences of these professionals in the face of changes and innovations in the process of caring for children and families during the COVID-19 pandemic, as well as understanding the typical experience of nurses in this care.

The “reasons why” experience showed that structural adjustments and changes in care constituted challenging aspects for these professionals. The reorganization of services was also observed in different pediatric care institutions^(16,17)^, as well as the difficulty in providing care, due to the high demand generated by COVID-19^(18)^.

Restrictions on the presence of visitors and companions, followed by adjustments in carrying out practices and procedures, were changes that occurred in the hospital environment. These policies were adopted in many institutions^(2)^ to respond to the risk of transmission of the new Coronavirus; however, the interruption of comprehensive care caused nurses to experience ethical challenges and moral suffering^(19,20)^.

In this process, aspects such as changes in clothing, precautions to avoid transmission of the virus, difficulty working with the new organization of the units, as well as difficulties and obstacles of various kinds were experienced by the group in question. Among the experiences experienced by front-line nurses during the pandemic in Qatar, it is described that the relocation of professionals to different units brought stress to these professionals^(21)^. Regarding clothing, there were divergences regarding the aspects found in this study, as the difficulty of use^(22,23)^ and the presence of facial injury^(24)^ were mainly highlighted.

Fear, lack of knowledge and greater exposure to the virus were also part of the participants’ trajectory. In this aspect, nurses draw attention to the impact on care, in addition to the loss of physical contact with peers, their families and hospitalized children and families, they highlighted that fear was reduced due to being vaccinated and working with children . Frontline nurses, at the beginning of the pandemic, did not have experience or information on how to protect themselves, thus reflecting greater concern among these professionals^(22)^. The fear of contaminating the family^(25)^ as well as the restriction of contact with family members were findings described in other realities^(21)^.

The use of coping strategies to continue care was also described by research participants, they were observed through self-care actions and peer support; these being aspects that helped with adaptation during the COVID-19 pandemic. The adaptation was reported in research with nurses who worked with patients affected by COVID-19 in China, through a sequence of three stages: the first was linked to the sense of professional mission and fear of the virus; the second, emotional exhaustion, and the third, psychological adaptation. The latter was related to familiarity with the environment and work processes, in addition to recognition and social support among peers^(23)^.

The adaptation stance was experienced by these professionals due to the perception that the worst phases of the pandemic were already over, as well as the adaptation to new work configurations. The last aspect deserves attention, since adaptation can mean the effective incorporation of practices that have been modified, especially those that concern the presence of family members in the hospital environment, thus harming patient- and family-centered care^(3)^.

Innovation in the ways of providing care represents the majority of actions carried out during this period. The nurses recognized alternatives for children to have access to toys, new forms of communication and technological means to establish communication with the family. The use of technology for care was the main measure recommended for health care during this health event^(7,8)^.

In addition to these measures, there was a need to evaluate and make restrictive visitation and monitoring routines more flexible. By making such routines more flexible, nurses put themselves in the family’s shoes, thus having repercussions on their work processes. For the phenomenological approach, all authentic understanding of the other is based on acts of self-interpretation by the subject who is dedicated to understanding. In this way, in this face-to-face relationship, established between nurses, children and families, the recovery of the baggage and knowledge of these professionals emerges^(10)^.

Despite the difficulties experienced, the changes brought by the pandemic brought learning. The exchange of experiences with professionals from another institution provided new knowledge. As a result, there was greater teamwork; aspect that corroborates other studies^(21,22)^. Reevaluating the care provided to the family was also a learning experience for nurses; the importance of parents as partners in care and the responsibility of the clinical team in encouraging alternatives to family presence were also aspects described^(17)^. Finally, the effective use of safety measures, another learning that occurred due to the high transmissibility, in addition to the contamination of colleagues and family members^(26)^.

The expectations regarding care and the work process, that is, the desire that nurses had to resume adequate care for children, with better working conditions, were aspects experienced that indicate their expectations for future projects, that is, the “reasons for” social action^(10)^.

In their experience, nurses demonstrated uncertainty about the end of the pandemic, however, they mentioned the desire for safe measures and appreciation for the work carried out. As for valorization, this has been a commonly highlighted aspect^(27)^. Projections of creative, humane and child- and family-centered measures represent an important expectation that converges with the standard of care in pediatrics, and which need to be ensured in times of crisis^(3)^.

## CONCLUSION

The essence of the phenomenon in the present study was evidenced in the changes brought about by the pandemic, the adaptation of the team, given the scenario of restrictions and risks, and the expectations given to the care of the child, the family and the work process. This experience brought learning, but also ethical dilemmas, due to the restrictions established for visitors and companions in the hospital environment, and moral suffering, for nurses in an attempt to guarantee child- and family-centered care.

## References

[1] Institute for Patient and Family Centered Care (2020). What is patient- and family-centered care?.

[2] Darcy Mahoney A, White RD, Velasquez A, Barrett TS, Clark RH, Ahmad KA (2020). Impact of restrictions on parental presence in neonatal intensive care units related to coronavirus disease 2019. J Perinatol.

[3] Hart JL, Turnbull AE, Oppenheim IM, Courtright KR (2020). Family-centered care during the COVID-19 era. J Pain Symptom Manage.

[4] Maaskant JM, Jongerden IP, Bik J, Joosten M, Musters S, Storm-Versloot MN (2021). Strict isolation requires a different approach to the family of hospitalised patients with COVID-19: a rapid qualitative study. Int J Nurs Stud.

[5] Aboumatar H (2020). Three reasons to focus on patient and family engagement during the COVID-19 pandemic. Qual Manag Health Care.

[6] Lemmon ME, Chapman I, Malcolm W, Kelley K, Shaw RJ, Milazzo A (2020). Beyond the first wave: consequences of COVID-19 on high-risk infants and families. Am J Perinatol.

[7] Aziz S, Arabi YM, Alhazzani W, Evans L, Citerio G, Fischkoff K (2020). Managing ICU surge during the COVID-19 crisis: rapid guidelines. Intensive Care Med.

[8] Gaulton J, Ziegler K, Chang E (2020). Virtual practices transform the care delivery model in an intensive care unit during the coronavirus pandemic. NEJM Catal.

[9] Morley G, Grady C, McCarthy J, Ulrich CM (2020). COVID-19: ethical challenges for nurses. Hastings Cent Rep.

[10] Schütz A (2018). A construção significativa do mundo social: uma introdução à sociologia compreensiva.

[11] Kirchherr J, Charles K (2018). Enhancing the sample diversity of snowball samples: recommendations from a research project on anti-dam movements in Southeast Asia. PLoS One.

[12] Minayo MC S (2017). Amostragem e saturação em pesquisa qualitativa: consensos e controvérsias. Revista Pesquisa Qualitativa.

[13] Kortchmar E (2018). A experiência de reganho de peso após a cirurgia bariátrica: um enfoque fenomenológico [doutorado].

[14] Tong A, Sainsbury P, Craig J (2007). Consolidated criteria for reporting qualitative research (COREQ): a 32-item checklist for interviews and focus groups. Int J Qual Health Care.

[15] Brasil. Ministério da Saúde (2012). Resolução n° 466, de 12 de dezembro de 2012. Dispõe sobre diretrizes e normas regulamentadoras de pesquisas envolvendo seres humanos.

[16] Somekh I, Somech R, Pettoello-Mantovani M, Somekh E (2020). Changes in routine pediatric practice in light of Coronavirus 2019 (COVID-19). J Pediatr.

[17] Tedesco B, Borgese G, Cracco U, Casarotto P, Zanin A (2020). Challenges to delivering family-centred care during the Coronavirus pandemic: voices of Italian paediatric intensive care unit nurses. Nurs Crit Care.

[18] Noronha KVMS, Guedes GR, Turra CM, Andrade MV, Botega L, Nogueira D (2020). Pandemia por COVID-19 no Brasil: análise da demanda e da oferta de leitos hospitalares e equipamentos de ventilação assistida segundo diferentes cenários. Cad Saúde Pública.

[19] Jia Y, Chen O, Xiao Z, Xiao J, Bian J, Jia H (2021). Nurses’ ethical challenges caring for people with COVID-19: a qualitative study. Nurs Ethics.

[20] Rezaee N, Mardani-Hamooleh M, Seraji M (2020). Nurses’ perception of ethical challenges in caring for patients with COVID-19: a qualitative analysis. J Med Ethics Hist Med.

[21] Villar RC, Nashwan AJ, Mathew RG, Mohamed AS, Munirathinam S, Abujaber AA (2021). The lived experiences of frontline nurses during the coronavirus disease pandemic in Qatar: a qualitative study. Nurs Open.

[22] Liu Y-E, Zhai Z-C, Han Y-H, Liu Y-L, Liu F-P, Hu D-Y (2020). Experiences of front-line nurses combating coronavirus disease-2019 in China: a qualitative analysis. Public Health Nurs.

[23] Zhang Y, Wei L, Li H, Pan Y, Wang J, Li Q (2020). The psychological change process of frontline nurses caring for patients with COVID-19 during its outbreak. Issues Ment Health Nurs.

[24] Coelho MMF, Cavalcante VMV, Moraes JT, de Menezes LCG, Figueirêdo SV, Branco MFCC (2020). Lesão por pressão relacionada ao uso de equipamentos de proteção individual na pandemia da COVID-19. Rev Bras Enferm.

[25] Góes FGB, Silva ACSSD, Santos ASTD, Pereira-Ávila FMV, Silva LJD, Silva LFD (2020). Desafios de profissionais de Enfermagem Pediátrica frente à pandemia da COVID-19. Rev Lat Am Enfermagem.

[26] Catania G, Zanini M, Hayter M, Timmins F, Dasso N, Ottonello G (2021). Lessons from Italian front-line nurses’ experiences during the COVID-19 pandemic: a qualitative descriptive study. J Nurs Manag.

[27] Conz CA, Braga VAS, Vasconcelos R, Machado FHRS, Jesus MCP, Merighi MAB (2021). Experiences of intensive care unit nurses with COVID-19 patients. Rev Esc Enferm USP.

